# Using the virtual reality device Oculus Rift for neuropsychological assessment of visual processing capabilities

**DOI:** 10.1038/srep37016

**Published:** 2016-11-21

**Authors:** Rebecca M. Foerster, Christian H. Poth, Christian Behler, Mario Botsch, Werner X. Schneider

**Affiliations:** 1 Neuro-cognitive Psychology, Bielefeld University, P. O. Box 100131, D-33501 Bielefeld, Germany; 2 Cluster of Excellence Cognitive Interaction Technology, Bielefeld University, P. O. Box 100131, D-33501 Bielefeld, Germany; 3 Computer Graphics and Geometry Processing, Bielefeld University,P. O. Box 100131, D-33501 Bielefeld, Germany.

## Abstract

Neuropsychological assessment of human visual processing capabilities strongly depends on visual testing conditions including room lighting, stimuli, and viewing-distance. This limits standardization, threatens reliability, and prevents the assessment of core visual functions such as visual processing speed. Increasingly available virtual reality devices allow to address these problems. One such device is the portable, light-weight, and easy-to-use Oculus Rift. It is head-mounted and covers the entire visual field, thereby shielding and standardizing the visual stimulation. A fundamental prerequisite to use Oculus Rift for neuropsychological assessment is sufficient test-retest reliability. Here, we compare the test-retest reliabilities of Bundesen’s visual processing components (visual processing speed, threshold of conscious perception, capacity of visual working memory) as measured with Oculus Rift and a standard CRT computer screen. Our results show that Oculus Rift allows to measure the processing components as reliably as the standard CRT. This means that Oculus Rift is applicable for standardized and reliable assessment and diagnosis of elementary cognitive functions in laboratory and clinical settings. Oculus Rift thus provides the opportunity to compare visual processing components between individuals and institutions and to establish statistical norm distributions.

Human interaction with the environment is usually guided by visual information. Most environments are complex, that is, they change rapidly and provide an abundance of visual information at any given moment. Everyday functioning requires that the changes in the visual environment are perceived on time so that actions can be controlled efficiently and flexibly^1^,^2^,^3^,^4^. This flexible action control depends on how fast visual objects can be processed^5^,^6^,^7^,^8^. Therefore, deficiencies in visual processing speed are one source of the declined everyday functioning in neurological and psychiatric disorders and in aging^9^,^10^,^11^,^12^,^13^. Reliable assessment of visual processing speed is a prerequisite for accurate diagnosis and, hence, for adequate treatment of all clinical conditions of impaired visual processing speed. This, however, bears a number of challenges that are not covered by state-of-the-art assessment techniques^14^.

Most current diagnostic assessments attempt to capture visual processing speed by measuring reaction time, that is, by measuring how fast individuals can respond to a visual object^15^,^16^,^17^,^18^. This has been criticized because reaction time is not only determined by the speed with which objects are processed but also by the speed of selecting and executing the motor response^19^. In clinical settings, this confound is of special importance: Many patient groups show motor impairments, which affect their reaction time but need not affect their visual processing speed. It is impossible to assess the visual processing speed of these patients from reaction-time-based assessments^10^,^19^.

One way to assess visual processing speed without confounds due to variations in motor performance is to give individuals unlimited time to respond, but to limit the presentation duration of visual objects instead. In this case, the key dependent variable is report accuracy rather than reaction time^20^. Such an approach to assess visual processing speed^9^,^10^,^19^ has been realized in diagnostic assessments grounding on Bundesen’s^1^ “theory of visual attention” (TVA). In this assessment procedure (Fig. 1), individuals briefly view letters, the presentation of which is terminated by pattern masks. Afterwards, the letters are to be reported, but without any speed requirements. Presentation durations are varied in order to enable assessing how letter recognition improves with increased presentation duration. If visual processing speed is high, individuals should be able to recognize the letters even at brief presentation durations (provided that the minimally required presentation duration is granted). In contrast, if processing speed is low, longer presentation durations should be required before the letters can be recognized. Therefore, the rate at which performance improves over presentation duration can be used as a measure of visual processing speed that is not confounded by motor abilities. Besides visual processing speed, two additional components of visual processing that are not available in reaction-time-based assessments can be obtained by using TVA-based diagnostic assessment^1^,^9^,^10^. The first component is the threshold of conscious perception. It measures the minimum time an object must be presented to allow visual processing for object recognition^1^. The second component is the capacity of visual working memory. Information about visual objects is retained in visual working memory for short periods of time. This enables their online perception, their use in action control, and their later recognition^21^,^22^,^23^,^24^. Thus, once processing of an object has been finished, it is encoded into visual working memory and can be acted upon, but only if visual working memory has enough remaining retention capacity^1^. It is important to note that the TVA-based visual processing components are well-grounded in cognitive neuroscience and experimental psychology research^25^. Therefore, they provide content validity in the sense that measurements are readily interpretable in terms of psychological processes of the human brain^19^.

In sum, diagnostics of visual processing capabilities based on limited stimulus presentation rather than on reaction time allow to characterize different components of processing of an individual while controlling for confounds due to motor control. Such an approach for visual assessment has been successfully developed within the TVA framework^1^ over the last 15 years^9^,^10^,^19^,^20^. However, the standard TVA-based assessment cannot circumvent other challenges that are inherent to clinical settings. Tests of visual processing are highly dependent on the exact visual assessment conditions^1^,^10^. These conditions include the specific monitor used, room lighting, the size of stimuli, and the head position and distance relative to the monitor. Reliable measurement of visual processing components requires that these conditions are highly standardized and psychophysically controlled. It is crucial that measurements are only comparable when they are obtained under the same visual assessment conditions. Measurements from different assessment setups are not comparable. Therefore, it is almost impossible to obtain statistical assessment norms that hold across different laboratories and clinics. As a consequence, visual processing components such as visual processing speed cannot be compared across various measurement conditions to inform clinical diagnosis.

How can we meet the requirement of highly standardized assessment conditions to ensure reliable measurement of visual processing components? A solution to this problem is provided by head-mounted displays for virtual reality applications, such as Oculus Rift (http://www.oculusvr.com/, Fig. 2). Assessing visual processing components using Oculus Rift allows to keep a number of factors constant that affect visual processing. Nuisance factors stemming from the use of different display settings, display technologies, and monitor models^26^,^27^,^28^,^29^,^30^ are controlled for by using identical assessment devices across institutions. Moreover, Oculus Rift provides a standardization of external visual assessment conditions. Factors such as room lighting and distance-to-screen are rendered ineffective by Oculus Rift because it covers the entire visual field, and is head-mounted. Constant presentation conditions can be realized easily in various assessment situations, because Oculus Rift is portable, lightweight, and works with standard PC hardware. In addition, it offers the possibility to examine patients who cannot be transported or cannot sit upright. Taken together, Oculus Rift offers promising solutions to current problems of assessing visual processing components in various clinical settings.

However, Oculus Rift differs from standard visual laboratory presentation conditions in a number of ways: The version available to us was the Development Kit 2 (DK2), which uses an OLED display with 75 Hz temporal resolution. This is in contrast to the 100 Hz CRT displays that are usually employed for visual testing. Such CRTs are preferred to standard 60 Hz LCD screens because they provide higher timing precision and allow short presentation durations^26^,^28^. Although the OLED technology employed in Oculus Rift is actually closer to a CRT than to an LCD, it still has to be established whether Oculus Rift grants sufficient reliability for assessing visual processing capabilities.

Here, we investigated whether Oculus Rift enables to assess visual processing components as reliably as the usually employed CRT screen – the standard for visual testing in the lab (Fig. 2). To this end, test-retest reliabilities of the TVA-based visual processing components, visual processing speed, capacity of visual working memory, and threshold of conscious perception were measured in the same participants for both, Oculus Rift and a CRT screen.

## Results

**Figure 1:**
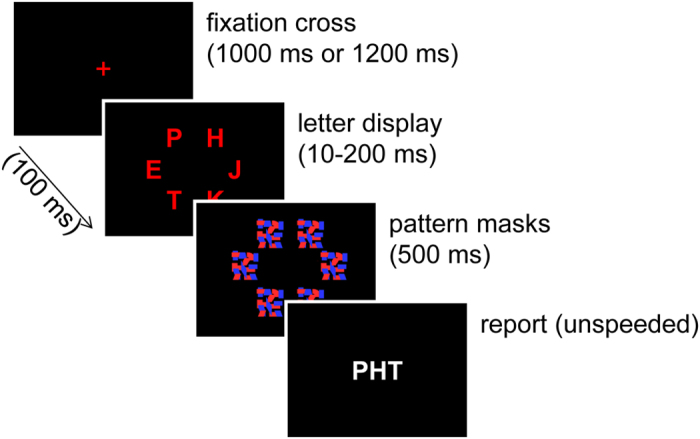
Trial sequence of the TVA-based assessment.

**Figure 2:**
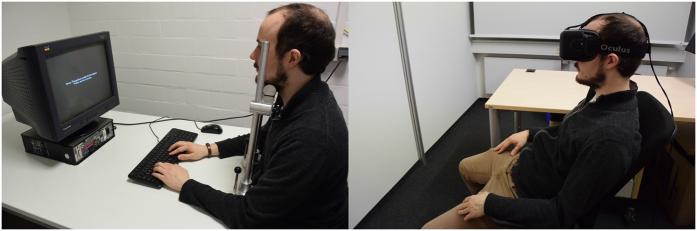
Assessment setups. A participant sitting in front of a standard CRT with head position fixed by a chin rest (left) and wearing the Oculus Rift virtual reality device (right).

Each participant completed the TVA-based assessment (Fig. 1) with a standard CRT screen and with Oculus Rift (Fig. 2) on a first day and repeated the two assessment types seven days later. Table 1 provides descriptive statistics of the resulting visual processing components, including the threshold of conscious perception, the capacity of visual working memory, and the visual processing speed. Measured processing components were significantly correlated between Oculus Rift and the CRT, both in the first and in the second session (see, Table 2 for correlations and *p*-values). Moreover, the threshold of conscious perception decreased from session 1 to session 2 for both devices (paired *t*-test with Cohen’s *d*_z_ as effect size; Oculus Rift: *t*(43) = 2.828, *p* = 0.007, *d*_z_ = 0.43; CRT: *t*(43) = 3.561 *p* < 0.001, *d*_z_ = 0.54). The capacity of visual working memory increased from session 1 to session 2 for Oculus Rift (*t*(43) = −2.224, *p* = 0.031, *d*_z_ = −0.34) but not for the CRT (*t*(43) = −0.348, *p* = 0.730, *d*_z_ = −0.05). Likewise, visual processing speed increased from session 1 to session 2 for Oculus Rift (*t*(43) = −6.286, *p* < 0.001, *d*_z_ = −0.95) but not for the CRT (*t*(43) = −1.068, *p* = 0.291, *d*_z_ = −0.16).

**Figure 3:**
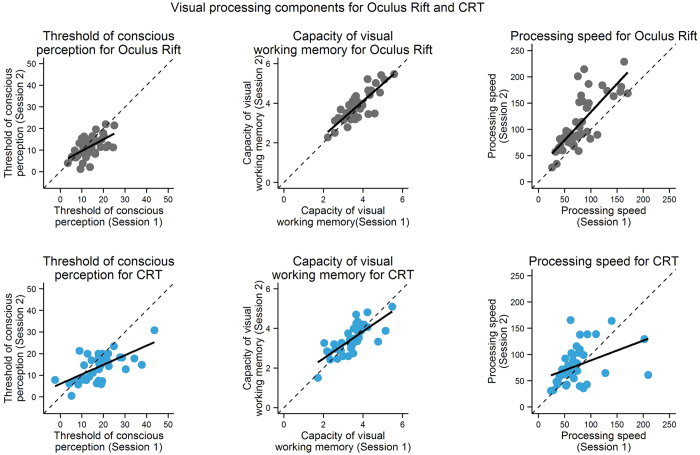
Test-retest reliabilities of visual processing components as linear regression lines along with the individual participants’ data. Threshold of conscious perception in ms (left column), capacity of visual working memory in the number of retained letters (middle column), and visual processing speed in letters/s (right column) of individual participants for both sessions of Oculus Rift (gray points, upper row) and CRT assessment (blue points, lower row). Values of session 1 are depicted on the x-axis, those of session 2 on the y-axis. The main diagonal indicates identical values for both sessions. Straight lines represent linear regressions.

Test-retest reliabilities of the three visual processing components were computed as Pearson’s product-moment correlations between participants’ components in the first and second session for each device. Figure 3 visualizes the test-retest reliabilities of all three visual processing components per assessment device as linear regression lines along with the individual participants’ visual processing components in the two sessions.

As can be seen in Table 3, the test-retest reliabilities of the three visual processing components were significant for both, Oculus Rift and the CRT. Steiger’s^31^ *Z*-test for independent correlations was used to compare test-retest reliabilities of Oculus Rift and the CRT. The test-retest reliabilities of the threshold of conscious perception (*Z* = 0.391, *p* = 0.696) and of the capacity of visual working memory (*Z* = 1.141, *p* = 0.254) did not differ significantly between Oculus Rift and the CRT. The reliability of visual processing speed was higher for Oculus Rift than for the CRT, *Z* = 2.550, *p* = 0.011. Because the reliability estimates might have been affected by outliers in the data (see Fig. 3), we repeated the analyses after excluding influential cases (see Supplementary Table S1 and Supplementary Fig. S1). This yielded the same pattern of results as the original analyses (see Supplementary Table S1).

Taken together, the reliability of Oculus Rift for assessing visual processing components (threshold of conscious perception, capacity of visual working memory, and visual processing speed) was *at least* as high as the reliability of the CRT.

## Discussion

Increasingly available virtual reality devices such as Oculus Rift offer great opportunities for the standardized assessment of visual processing components and neuropsychological diagnostics. Oculus Rift covers the entire visual field and excludes nuisance factors stemming from external testing conditions. Thereby, it offers an inherent standardization of the visual stimulation, which is a crucial requirement for the neuropsychological assessment of visual processing components. With its portability, Oculus Rift enables standardized measurements of visual processing components that do not depend on laboratory setups and can be performed under real-life clinical conditions. This paves the way for comparing measurements between patients and institutions and to establish statistical norm distributions. It also enables to assess patients with impaired motor skills and those who cannot sit up, and would otherwise not be examinable.

A fundamental prerequisite for using Oculus Rift to assess visual processing components is that it provides sufficient reliability. Here, we investigated whether Oculus Rift allows to measure visual processing components as reliable as standard measurement setups. To this end, we compared the test-retest reliabilities of visual processing components measured using Oculus Rift and measured using a CRT screen, the current gold-standard. We found that the test-retest reliability of Oculus Rift was comparable to the standard CRT assessment procedure for all three visual processing components: the threshold of conscious perception, the capacity of visual working memory, and the speed of visual processing. As such, the present findings provide a first step towards the neuropsychological assessment of visual processing capabilities using head-mounted virtual reality devices.

We investigated visual processing components by using the TVA-based assessment procedure^9^,^10^ which is based on Bundesen’s theory of visual attention^1^. For healthy participants, the standard assessment procedure uses a CRT screen and requires participants to report letters by entering them on a keyboard^13^. This procedure has been used in a number of studies with healthy participants as well as in a previous reliability study^14^. Oculus Rift covers the entire visual field so that participants cannot view the keyboard. Therefore, in the Oculus Rift assessment, participants reported letters verbally to the experimenter who typed them in on the keyboard. Such a verbal report procedure is a common way to accommodate problems of typed letter reports, for instance due to motor impairments of clinical patients^32^. To prevent the different report procedures from affecting the results, in both assessments, participants viewed reported letters on screen, could correct their reports, and had to confirm them before continuation. The methodic difference between Oculus and CRT report might limit the exact comparability of the two assessments. It is important to note, however, that the aim of the present study was to establish if it is possible to measure visual processing components using Oculus Rift with sufficient reliability. Our results show that this is the case, even if the components had been affected by the report procedure.

Oculus Rift seems to assess visual processing components with a test-retest reliability that is comparable to that of a standard CRT. However, we also found that the mean visual processing components across participants changed from the first to the second session, which is in line with earlier findings^14^. While the threshold of conscious perception decreased from session 1 to session 2 for both devices, the capacity of visual working memory and visual processing speed increased only for Oculus Rift. These findings might reflect practice effects that are more pronounced for Oculus Rift than for the CRT. This has to be taken into account for repeated assessments of the same participants (cf.^14^).

Virtual reality has been used previously in clinical neuropsychology^33^,^34^,^35^,^36^,^37^,^38^,^39^,^40^,^41^,^42^. However, these studies focused on exhausting the presentation formats provided by virtual reality such as 360° three-dimensional stimulation with ecological validity, controlled interaction scenarios, and the possibility to change the environment and agent embodiment online. However, to the best of our knowledge, no study investigated whether virtual reality can be used for reliable neuropsychological diagnostics of visual processing capabilities.

In sum, the head-mounted virtual reality device Oculus Rift is applicable for the standardized and reliable assessment of visual processing components in the laboratory as well as in clinical settings. It provides the opportunity to compare visual processing components between individuals and institutions and to establish statistical norm distributions. The present experiment was based on the latest developer-version (DK2) of Oculus Rift. The final consumer version is available now (time of publication) at a price of about 700 Euro and provides an even better display hardware with a refresh rate of 90 Hz (11.1 ms per frame) and a spatial resolution of 1,080 × 1,200 pixels per eye. This update should eliminate the slight technical disadvantages of the DK2 prototype compared to 100 Hz CRT screens, such that we can expect even better results with the consumer version hardware. To allow researchers and practitioners to reproduce our results, we will make our Oculus-TVA-software publicly available at http://www.uni-bielefeld.de/psychologie/ae/Ae01/Research/VR/.

## Methods

Forty-eight participants were recruited at Bielefeld University, Germany, and all provided written informed consent before the start of the experiment. All participants reported normal or lens-corrected visual acuity, were naïve with respect to the purpose of the study, and were paid for participation. Two participants did not complete all experimental sessions and were excluded from the analysis. Two further participants were not included in the analysis because they did not comply with task instructions. The exclusion of these participants did not change the overall result pattern. The remaining sample of 44 participants consisted of 27 males and 17 females with an average age of 24 years (ranging from 18 to 39). Thirty-four participants were right-handed and 10 were left-handed. The study was approved by the Bielefeld University’s ethics committee and performed in accordance with the approved guidelines.

TVA-based assessment was performed with a CRT screen as well as with Oculus Rift (Fig. 2). CRT assessment was controlled by the E-Prime software (version 2.0). The stimuli were displayed on a 19-inch CRT color monitor (G90fB, ViewSonic, Brea, CA, USA) with a refresh rate of 100 Hz and a spatial resolution of 1,024 × 768 pixels extending 36 × 27 cm. Participants’ heads were stabilized by a chin rest at a viewing distance of 71 cm from the CRT screen.

Assessment with Oculus Rift was performed using the version “Development Kit 2” (DK2). This device features a low-persistence OLED display with a refresh rate of 75 Hz and a spatial resolution of 1,920 × 1,080 pixels. Because Oculus Rift is targeted at stereoscopic visualization for virtual reality applications, the display is split horizontally, and individual images for the left/right eye are shown on the left/right half of the display, resulting in a resolution of 960 × 1,080 pixels for each eye. The impressive field of view of about 111 degrees is achieved by placing two fisheye lenses in front of the left and right eye, which magnify (and distort) the views onto the left/right image. The visualization software properly transforms the rendered images using the inverse lens distortion, such that both distortions cancel out and the final image looks normal again. Because of this mechanism, the spatial resolution of Oculus Rift cannot be directly compared to the CRT, and in fact the perceived resolution is lower than that of the CRT. The special low persistence OLED screen has an individual light source for every pixel. During one time frame (13.3 ms for 75 Hz refresh rate), pixels are illuminated in a sweep from left to right, such that each pixel is excited for about 2 ms only. This technique, which was designed to minimize motion blur in virtual reality applications, makes Oculus Rift behave very similar to CRT screens, where a sweeping cathode-ray excites pixels for a similarly short period of time^28^.

All stimuli were displayed on a black background. A red plus (11 pixels = 0.31 degrees of visual angle [°v.a.]) served as central fixation cross. A set of 20 red capital letters (ABDEFGHJKLMNOPRSTUVX), written in bold Arial font (font size of 68 points, corresponding to about 1.87°v.a. in height and 1.22–1.79°v.a. in width), were displayed for later report. At each trial, six red letters were presented at 45°, 90°, 135°, 225°, 270°, and 315° of an imaginary circle around the central fixation cross (180 pixels = 5.10°v.a. radius). Each letter was followed by one of eight different red-blue pattern masks (100 × 100 pixels = 2.84 × 2.84°v.a.).

Due to the 3D stereoscopic visualization and the fisheye lens distortion in Oculus Rift, the effective sizes, distances, and viewing angles of presented visual stimuli (after lens distortion) cannot be determined exactly from the stimulus’ pixel sizes and positions before lens distortion. For all stimuli, we therefore manually adjusted their respective sizes, screen positions, and viewing parameters, such that the perceived visual stimuli in Oculus Rift matched those in the CRT setup as closely as possible.

Participants completed two TVA-based assessment sessions with each device (CRT and Oculus Rift) separated by 7 days at about the same time of day. On the first assessment day, participants completed a first session with each device. On the second assessment day, participants completed the second session of each device. Assessment lasted for about one hour each day. The four possible assessment orders varied across participants (Table 4).

A modified version of the standard TVA-based assessment from Vangkilde *et al*.^43^ was used (see Fig. 1). The TVA-based assessment consisted of 9 blocks of 18 whole report trials per block preceded by a block of 18 practice trials. Each trial started with a red central fixation cross. Then, six red letters were presented on an imaginary circle around the fixation cross. The letters were randomly chosen on each trial, with each letter appearing only once. Each letter was followed by a red-blue pattern mask lasting for 500 ms. Each of the six masks for an individual trial was randomly chosen from the set of pattern masks without replacement. Participants were instructed to keep central fixation throughout the presentation. Afterwards, the screen went black and participants had to give an unspeeded report of all letters they remembered. During CRT assessment, they typed the letters in any order on a standard keyboard. This report procedure has been used in several CRT-based TVA studies with healthy participants^6^,^43^ and also in the prior TVA reliability study^14^. During Oculus Rift assessment, participants reported the letters verbally in any order and the experimenter typed them in. Verbal report was necessary during Oculus Rift assessment as participants could neither view the keyboard, nor their hands while wearing the head-mounted device (see Fig. 2).

Stimulus durations varied systematically. During CRT assessment, the letters were shown for 10, 20, 50, 80, 140, or 200 ms, which are all multiples of the single-frame-time of 10 ms (at 100 Hz refresh rate). The fixation cross lasted for 1,200 ms followed by a 100 ms blank. Oculus Rift has a lower refresh rate of 75 Hz, corresponding to a single-frame time of 13.3 ms. The presentation durations of Oculus assessment were therefore chosen to be the multiples of 13.3 ms that best matched the CRT times: 13, 27, 53, 80, 147 or 200 ms. The fixation cross lasted for 1000 ms.

Participants were instructed to report the letters they were fairly certain of having seen but to refrain from guessing^43^. Specifically, they were to aim at an accuracy of reported letters between 80 and 90% (i.e., at error rates between 20 and 10%; note that this measure only refers to typed-in letters so that reporting no letter does not count as an error). They were informed about their report accuracy after each block and they were reminded of the accuracy range. Participants’ error rates were in accordance with the instructed range (Oculus Rift: Session 1: *M* = 0.10, *SD* = 0.04, Session 2: *M* = 0.10, *SD* = 0.05; CRT: Session 1: *M* = 0.13, *SD* = 0.08, Session 2: *M* = 0.12, *SD* = 0.06).

Implementing the visualization for Oculus Rift assessment is relatively straightforward using the publicly available software development kit (SDK version 0.4.3 in our case). The image distortion process, however, requires special attention, because a naïve implementation might not be efficient enough to guarantee frame timings of 13.3 ms. Once a single frame takes longer than 13.3 ms to compute, for instance 15 ms, it will only be displayed at the next screen refresh, i.e., after 26.6 ms. This could for example prolong the presentation duration of the letters for one frame of 13.3 ms, which should noticeably influence participants’ performance in reporting the letters. In our experience, this can easily happen when Oculus Rift is connected to a less powerful laptop computer instead of a high performance workstation or gaming PC. A less powerful laptop computer can take too long to compute the images for Oculus Rift within the required time frame because the image computation routines are not fast enough. In the following, we present a very efficient method that yields sufficiently high frame rates even for image computations made on low-performance laptops, which might be used in clinical settings (bedside testings).

**Figure 4:**
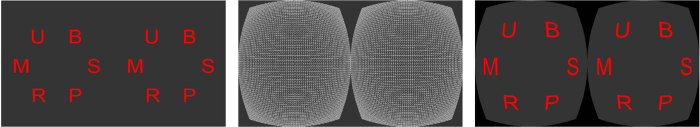
Distortion process for Oculus Rift. After the images for the left and right eye are rendered (left), they are pasted as textures onto the distortion meshes (center), leading to the final distorted images (right), which appear normal when viewed through the fisheye lenses of Oculus Rift. Note that the letters are rendered much larger than in our Oculus-TVA application to better visualize the distortion effect.

Visualizing the stimuli on Oculus Rift is a three-step process (see Fig. 4). First, the scene is rendered as seen from the left eye and stored into the left half of the framebuffer. In a second pass, the right half of the screen is filled with the right eye’s view. These two render passes result in the image shown in Fig. 4, left. In the third pass, the two images are distorted in order to compensate for the effect of the fisheye lenses. This is done by mapping the undistorted images as textures onto a special, pre-computed ‘distortion mesh’ (Fig. 4, center), which only depends on the (known and constant) lens parameters and is provided by Oculus SDK. The result is the distorted image shown on the right side in Fig. 4, which looks normal and undistorted when seen through the fisheye lenses. For the first two render passes, the six red letters or red-blue masks are visualized as rectangles (i.e., two triangles each) with the corresponding letter/mask texture mapped onto them. Rendering these 12 textured triangles is highly efficient. The distortion meshes for the left and right eye consist of 8,192 triangles each, such that in the third render pass 16,384 triangles have to be rendered for the textured distortion meshes of the left and right eye, which is much more expensive, requires about 90% of the computation time, and might extend the available 13 ms frame time.

In our implementation, we exploit the fact that most of the TVA stimuli do not change each frame, and thus can be precomputed. Instead of letting Oculus SDK distort the images and immediately display them on Oculus Rift, we perform the image distortion ourselves and store the resulting distorted image in a 2D texture. In this manner, we precompute the distorted images (Fig. 4, right) for the center fixation cross as well as for the red-blue masks at the application start, because those do not change during the assessment. Due to the fact that the sets of six letters are chosen randomly in TVA assessment, we can neither precompute them at the application start, nor can we precompute all possible letter combinations due to GPU memory restrictions. We can, however, easily precompute the distorted TVA letter image for each individual trial during the presentation of the fixation cross (1,000 ms) preceding that trial. After these precomputations, all visual stimuli (fixation cross, letters, and masks) are displayed on Oculus Rift at almost no computational cost. With this simple but effective technique we could reliably perform even the time-critical short presentation durations of 13 ms and 26 ms on a low-performance laptop.

The three components of visual processing (threshold of conscious perception, capacity of visual working memory, and visual processing speed) were obtained for each participant and each assessment by fitting the number of correctly reported letters with an ex-Gaussian distribution^44^ (see below), which is a generalization of the classic exponential TVA model^1^. Fitting was accomplished by using a maximum-likelihood fitting procedure provided by the LIBTVA toolbox^44^ for MATLAB (R2013b, The Mathworks, Natick, MA, USA; see also ref. 45). The three components of visual processing are based on the following assumptions^1^. Visual processing of objects starts once a minimum presentation duration of the objects (here the presentation duration of the letters in ms) has been reached. This duration is the *threshold of conscious perception* and it is assumed to vary across trials following a normal distribution with a given mean and standard deviation (two free parameters).

Visual processing consists in making categorizations of objects, for example, in categorizing a letter as being an R. Reporting the categorization of an object requires that the categorization has been encoded into visual working memory. Visual working memory can retain all categorizations of only a limited number of objects and this defines its capacity in terms of distinct objects^1^. The *capacity of visual working memory* (here measured in the number of retained letters) is assumed to vary across trials according to a probability distribution with five free parameters, the probability that the capacity is 1, 2, …, 5. The probability that the capacity is 6 equals 1 minus the sum of the five parameters. The capacity of visual working memory is then estimated as the expected value of this distribution. Categorizations of objects that finish processing first are encoded into visual working memory but only until memory is filled up with categorizations of different objects. In the classic TVA-model^1^, the processing times of individual object categorizations are assumed to be exponentially distributed (after the threshold of conscious perception has been reached). Thus, the rate parameters of these exponential distributions reflect the processing speed of the categorizations. The sum of the rate parameters reflects the overall available visual processing capacity and equivalently the total *visual processing speed*, which is assumed to be constant (one free parameter). Here, we used a refinement of the classic TVA model by Dyrholm *et al*.^44^, in which the processing times of object categorizations are assumed to follow an ex-Gaussian distribution (i.e., the convolution of an exponential and a Gaussian distribution^46^) rather than an exponential distribution due to the normal distribution of the threshold of conscious perception. Besides the three components of visual processing, participants’ error rates (rates of erroneously reported letters) were assessed to check whether participants were in the instructed accuracy range between 80 and 90%.

## Additional Information

**How to cite this article**: Foerster, R. M. *et al*. Using the virtual reality device Oculus Rift for neuropsychological assessment of visual processing capabilities. *Sci. Rep.* **6**, 37016; doi: 10.1038/srep37016 (2016).

**Publisher’s note**: Springer Nature remains neutral with regard to jurisdictional claims in published maps and institutional affiliations.

## Supplementary Material

Supplementary Information

## Figures and Tables

**Table 1:** Descriptive statistics. Means (*M*) and standard deviations (*SD*) of visual processing components (threshold of conscious perception in ms, processing speed in letters/s, and capacity of visual working memory in the number of retained letters) for the two sessions performed using Oculus Rift and the two sessions performed using the CRT.

	Oculus Rift		CRT	
	Session 1	Session 2	Session 1	Session 2
	*M* *(SD)*	*M* *(SD)*	*M* *(SD)*	*M* *(SD)*
Threshold of conscious perception	13.37 (5.18)	11.42 (4.76)	17.41 (8.89)	13.70 (6.20)
Capacity of visual working memory	3.71 (0.71)	3.85 (0.74)	3.43 (0.76)	3.45 (0.69)
Processing speed	81.17 (36.54)	112.83 (51.38)	72.01 (37.68)	78.34 (34.57)

**Table 2:** Correlations (Pearson’s *r*) between measurements of the two assessment devices Oculus Rift and CRT for each of the three visual processing components (threshold of conscious perception, visual processing speed, and capacity of visual working memory) per session.

	Session 1	Session 2
	*r* (*p*)	*r* (*p*)
Threshold of conscious perception	0.37 (0.014)	0.54 (<0.001)
Capacity of visual working memory	0.72 (<0.001)	0.77 (<0.001)
Processing speed	0.51 (<0.001)	0.66 (<0.001)

*p*-values are shown in parentheses.

**Table 3:** Test-retest reliabilities of the three visual processing components (threshold of conscious perception, visual processing speed, and capacity of visual working memory) for Oculus Rift and CRT.

	Oculus Rift		CRT	
	*r* (*p*)	CI of *r*	*r* (*p*)	CI of *r*
Threshold of conscious perception	0.58 (<0.001)	[0.34; 0.75]	0.63 (<0.001)	[0.41; 0.78]
Capacity of visual working memory	0.84 (<0.001)	[0.72; 0.91]	0.74 (<0.001)	[0.58; 0.85]
Processing speed	0.76 (<0.001)	[0.60; 0.86]	0.41 (0.006)	[0.13; 0.63]

Test-retest reliabilities are provided as Pearson’s *r* and along with associated *p-*values and confidence intervals (CI).

**Table 4:** Number of participants per assessment order.

Number of participants	Day 1	Day 2
15	Oculus first	Oculus first
11	CRT first	CRT first
7	Oculus first	CRT first
11	CRT first	Oculus first
