# Erratum: Using the virtual reality device Oculus Rift for neuropsychological assessment of visual processing capabilities

**DOI:** 10.1038/srep41464

**Published:** 2017-02-15

**Authors:** Rebecca M. Foerster, Christian H. Poth, Christian Behler, Mario Botsch, Werner X. Schneider

Scientific Reports 6: Article number: 3701610.1038/srep37016; published online: 11 21 2016; updated: 02 15 2017

The original version of this Article contained referencing errors, where multiple instances of reference 5 were incorrectly given as reference 1. The publishers regret introducing these errors during final typesetting.

This has now been corrected in the HTML and PDF versions of this Article.

