# Supplementary material for: Using the virtual reality device Oculus Rift for neuropsychological assessment of visual processing capabilities
Source: Sci Rep. 2016 Nov 21;6:37016. doi: 10.1038/srep37016 (PMC5116630; doi:10.1038/srep37016)
Supplement: Supplementary Information [file srep37016-s1.pdf]

Supplementary information

Using the virtual reality device Oculus Rift for neuropsychological assessment of visual processing capabilities

Rebecca M. Foerster[1,3,*], Christian H. Poth[1,3,*], Christian Behler[2,3], Mario Botsch[2,3], & Werner X. Schneider[1,3]

[1]Neuro-cognitive Psychology, Bielefeld University, Bielefeld, Germany

[2]Computer Graphics and Geometry Processing, Bielefeld University, Bielefeld, Germany

[3]Cluster of Excellence Cognitive Interaction Technology, Bielefeld University, Bielefeld, Germany

*shared first authorship

Correspondence to: Rebecca M. Foerster (rebecca.foerster@uni-bielefeld.de) or Christian H. Poth (c.poth@uni-bielefeld.de)

Supplementary Table S1. Test-retest reliabilities of the three visual processing components (threshold of conscious perception, visual processing speed, and capacity of visual working memory) for Oculus Rift and CRT after influential cases (outliers) had been excluded separately for each individual processing component (i.e., cases that were excluded for one component were included in analyses of the other components). Individual cases were diagnosed as influential (outliers) by using the influence measures for linear models provided by the *stats* package for R[1]. Cases were diagnosed as influential if any of the following measures exceeded the standard boundaries (stated in parentheses): DFBeta ($|DFBeta|$ > 1), DFFit ($|DFFit|$ > $3 \times \sqrt{k/(n-k)}$), the covariance ratio ($|1 - covariance\ ratio|$ > $3 \times k/(n-k)$), Cook's distance (Cook's distance > the 50% percentile of an F-distribution with k and $n-k$ degrees of freedom), or the leverage value (leverage value > $3 \times k/n$), whereby k is the number of model parameters of the linear regression, i.e. k = 2, and n is the sample size. Test-retest reliabilities are provided as Pearson's *r* and with associated *p*-values and confidence intervals (CI). Steiger's[2] *Z*-test for independent correlations was used to compare test-retest reliabilities of Oculus Rift and the CRT, and *Z* and *p*-values for these tests are provided as well.

	**Oculus Rift**		**CRT**		**Oculus Rift vs. CRT**	
	r (*p*)	CI of *r*	*r* (*p*)	CI of *r*	*Z*	*p*
Threshold of conscious perception	.58 (< .001)	[.33; .75]	.61 (< .001)	[.37; .77]	0.187	.852
Capacity of visual working memory	.86 (< .001)	[.74; .92]	.74 (< .001)	[.55; .86]	1.384	.166
Processing speed	.81 (< .001)	[.66; .89]	.38 (.020)	[.06; .62]	3.052	.002

Supplementary Figure S1. Test-retest reliabilities of visual processing components as linear regression lines along with the individual participants' data. Two regression lines are shown for each component. Black straight lines illustrate the regression by using all of the data (as reported in the main article). Green straight lines illustrate the regression after the exclusion of influential cases (outliers, orange data points). Individual cases were diagnosed as influential using the influence measures for linear models provided by the stats package for R[1] (using the criteria stated above, in the description of Supplementary Table S1). Depicted are the threshold of conscious perception in ms (left column), the capacity of visual working memory in the number of retained letters (middle column), and the visual processing speed in letters/s (right column) of individual participants for both sessions of Oculus Rift (gray points, upper row) and CRT assessment (blue points, lower row). The values of session 1 are depicted on the x-axis, those of session 2 on the y-axis. The main diagonal indicates identical values for both sessions.

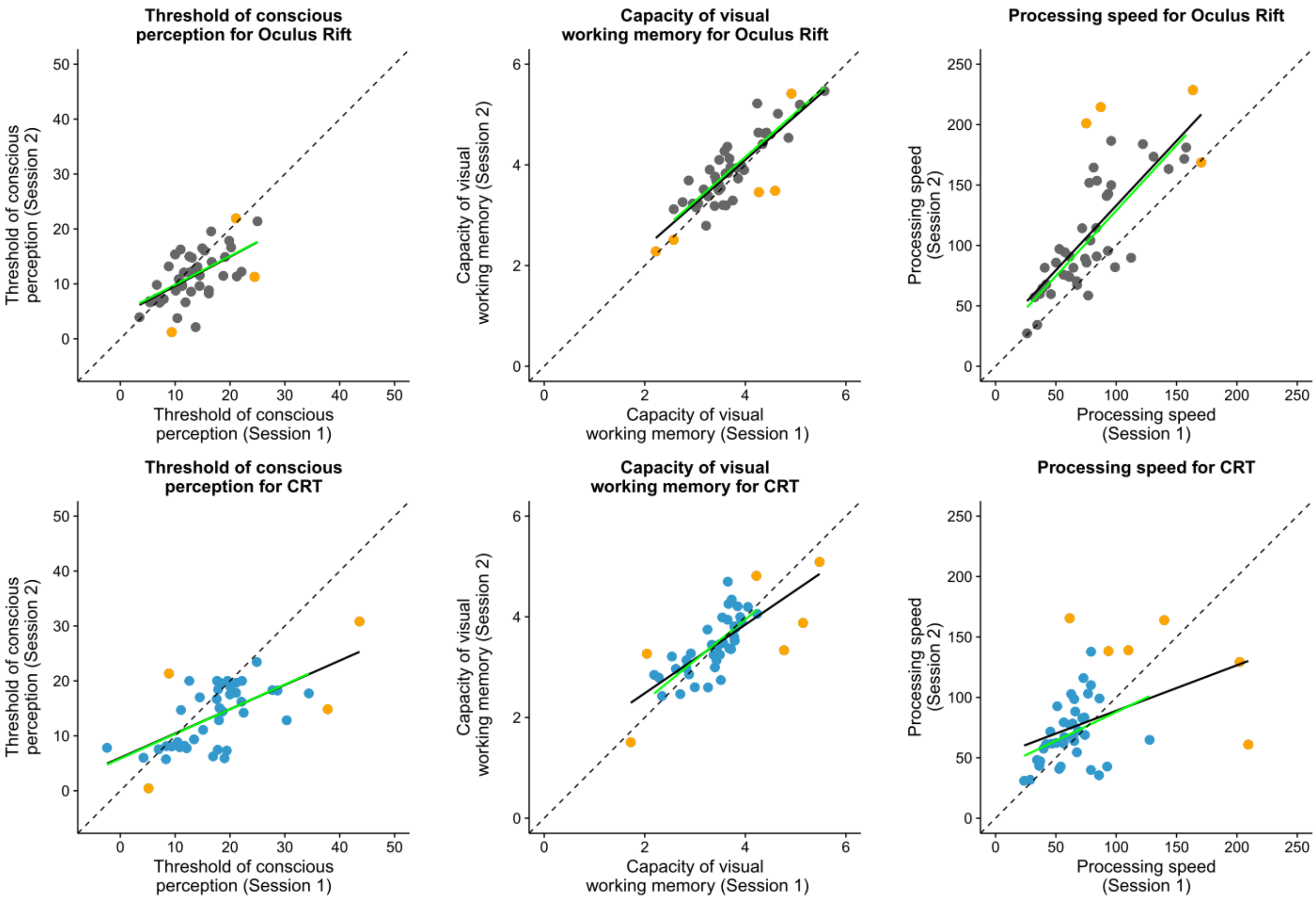

Supplementary References

1. R Core Team (2016). R: A language and environment for statistical computing. *R Foundation for Statistical Computing*, Vienna, Austria. https://www.R-project.org/.
2. Steiger, J. H. Tests for comparing elements of a correlation matrix. *Psychol. Bull.* **87**, 245–251 (1980).
